# Manufacturing Economics of Plant-Made Biologics: Case Studies in Therapeutic and Industrial Enzymes

**DOI:** 10.1155/2014/256135

**Published:** 2014-05-29

**Authors:** Daniel Tusé, Tiffany Tu, Karen A. McDonald

**Affiliations:** ^1^Intrucept Biomedicine LLC, 2695 13th Street, Sacramento, CA 95818, USA; ^2^Department of Chemical Engineering and Materials Science, University of California, 1 Shields Avenue, Davis, CA 95616, USA

## Abstract

Production of recombinant biologics in plants has received considerable attention as an alternative platform to traditional microbial and animal cell culture. Industrially relevant features of plant systems include proper eukaryotic protein processing, inherent safety due to lack of adventitious agents, more facile scalability, faster production (transient systems), and potentially lower costs. Lower manufacturing cost has been widely claimed as an intuitive feature of the platform by the plant-made biologics community, even though cost information resides within a few private companies and studies accurately documenting such an advantage have been lacking. We present two technoeconomic case studies representing plant-made enzymes for diverse applications: human butyrylcholinesterase produced indoors for use as a medical countermeasure and cellulases produced in the field for the conversion of cellulosic biomass into ethanol as a fuel extender. Production economics were modeled based on results reported with the latest-generation expression technologies on *Nicotiana* host plants. We evaluated process unit operations and calculated bulk active and per-dose or per-unit costs using SuperPro Designer modeling software. Our analyses indicate that substantial cost advantages over alternative platforms can be achieved with plant systems, but these advantages are molecule/product-specific and depend on the relative cost-efficiencies of alternative sources of the same product.

## 1. Introduction

This study represents original research on the manufacture of plant-made biologics (PMB) and plant-made industrial products (PMIP) through application of analytical modeling tools* in silico*. The main goal of this study was to evaluate unit operations in two plant-made biomanufacturing processes and estimate the cost of goods of the active ingredient (AI) and the impact of those costs on the cost of the final product. A secondary but equally important goal was to compare the manufacturing cost of plant-produced AI to the cost of the same AI manufactured by predecessor technologies.

Much progress has been made towards the development of manufacturing infrastructure for plant-made pharmaceuticals (PMP), which typically consist of recombinant proteins applied as vaccine antigens, therapeutic enzymes, or monoclonal antibodies. Progress has also been made in the manufacture of plant-based biologics, biochemicals, and biomaterials for industry, food, and other applications. Significant and industrially relevant advances in gene expression and bioprocessing methods have been achieved during the past two decades, as reviewed in several prior studies [1–7]. Yet, to date, only three PMP products have been approved by regulatory agencies for commercial sale, including an anti-caries antibody (Planet Biotechnology, USA), an animal health vaccine (Dow AgroSciences, USA), and a therapeutic enzyme to manage a metabolic disorder (Protalix Biotherapeutics, Israel) [5]. This relative scarcity of PMP products reflects the magnitude of the challenges in creating a new manufacturing industry. The development of the plant-based platform has slowly progressed through a multinational “labor of love” in the absence of the levels of investment originally made by the biopharmaceutical industry (with significant help from the venture capital community), which resulted in elevation of fermentation-based systems to their current level of dominance.

Interestingly, beginning in 2009, the US Defense Advanced Research Projects Agency's (DARPA) Blue Angel program made several multimillion dollar investments at various sites with the goals of accelerating the scale-up of the PMP infrastructure and assessing production of relevant volumes of pandemic influenza candidate antigens as a model product to test the plant-based platform (http://www.darpa.mil/NewsEvents/Releases/2012/07/25.aspx). This was a shared investment initiative, and as a result of federal and state government and private investments, the expanded PMB manufacturing capacity should now support production of at least several of the many plant-made vaccines, biotherapeutics, biomaterials, and biocatalysts that are under development by companies and institutions worldwide (most recently reviewed by Gleba et al. [5]). Although capacity expansion helped companies that would manufacture their own or partnered products (e.g., Caliber Biotherapeutics, Bryan, Texas, USA; Medicago Inc., Research Triangle Park, North Carolina, USA), these investments also helped expand capacity at PMP contract development and manufacturing organizations (CDMO) such as Kentucky BioProcessing (Owensboro, Kentucky, USA). This was important to our modeling because the decision to construct a new dedicated manufacturing facility versus contracting services from a CDMO could yield very different cost-of-goods projections.

Fundamental to the commercial introduction of PMB products is the availability of an efficient plant-based manufacturing infrastructure that is at a minimum competitive with and ideally superior to traditional animal cell and microbial fermentation systems as well as to extraction from raw materials from natural sources. The cost to manufacture any product is of paramount importance to its market acceptability, availability to those who need it most, and to the profitability of the product for its manufacturer. While plant-based technologies are often assumed to offer significant cost advantages relative to cell-based fermentation, such assumptions are based on the lower upstream capital investments required for plant growth, lower cost of media, no adventitious agent removal, and other factors [8–13]. However, few of these studies have listed engineering process assumptions or analyzed unit operations adequately; reports such as those of Evangelista et al. [14] and Nandi et al. [15] are exceptions. Therefore, results of recent technoeconomic evaluations for PMP/PMB/PMIP have not been widely available in the public literature.

To analyze and quantify the cost efficiency of plant-based manufacturing, we chose two enzymes representing active ingredients (AI) for diverse product classes and derived for each AI the bulk product (i.e., bulk active) and per-dose or per-unit costs. The first target analyzed is human butyrylcholinesterase (BuChE), an enzyme that can act as a bioscavenger to counteract the effects of cholinesterase inhibitors such as sarin and that is a candidate for biodefense countermeasures in several countries. While this product would encounter market dynamics that are different from other commercial products, it is nevertheless designed to satisfy an important component of public safety and merits review. Currently, BuChE is extracted from outdated human blood supplies, but it can also be made recombinantly in cell culture, transgenic animals, and plant systems.

The second case study focuses on the cellulase complex, a mixture of 4–6 enzymes used to saccharify cellulosic feedstocks for the production of ethanol as a fuel extender. This target was selected for study because, for more than 30 years, the cost of cellulases has been a major impediment to the economic viability of cellulosic ethanol programs. Cellulases were also selected because they represent an extremely cost-sensitive product class on which to conduct case studies. We reasoned that if plant-based manufacturing showed economic promise for this class, then the economically advantageous production of less cost-sensitive biotherapeutics and other products might also be anticipated. In contrast to BuChE, which consists of a purified molecule, the cellulase complex would be expressed in plants that are cultivated near the cellulosic feedstock and the bioethanol refinery and stored as silage without purification; the semidried catalyst biomass is mixed on demand with the cellulosic feedstock to initiate saccharification followed by fermentation. This approach varies significantly from previous approaches in which cellulase enzymes are produced via fermentation processes using native or engineered microorganisms. For the cellulase case study, the plant-based cellulase production process is compared with a recent technoeconomic analysis of cellulase enzymes produced from* Trichoderma reesei* fermentation using steam-exploded poplar as a nutrient source [16].

## 2. Materials and Methods

### 2.1. Modeling Software

The technoeconomic modeling for both case studies was performed using SuperPro Designer, Version 9.0 (Intelligen, Inc., Scotch Plains, NJ; http://www.intelligen.com/), a software tool for process simulation and flowsheet development that performs mass and energy balances, equipment sizing, batch scheduling/debottlenecking, capital investment and operating cost analysis, and profitability analysis. This software has been used to estimate cost of goods in a variety of process industries including pharmaceuticals produced by fermentation [17] and plant-made pharmaceuticals [14, 18]. It is particularly useful at the early, conceptual plant design stage where detailed engineering designs are not available or warranted. SuperPro Designer was chosen because it has built-in process models and an equipment cost database for typical unit operations used in the biotechnology industry, such as bioreactors, tangential flow ultrafiltration and diafiltration, chromatography, grinding/homogenization, and centrifugation. There are some unit operations and processes used in the case studies that are currently not included in SuperPro Designer, such as indoor or field plant cultivation, plant harvesting, vacuum agroinfiltration, and screw press/disintegrator. For the butyrylcholinesterase case study, SuperPro Designer's “Generic Box” (bulk flow, continuous) unit procedure was used to model these unit operations. For the cellulase case study, the indoor unit operations were modeled with the same software while the field production calculation and costs were tracked in Microsoft Excel spreadsheets. Unless otherwise noted, the costs of major equipment, unit operation-specific labor requirements and costs (e.g., operators, supervisors), pure components, stock mixtures, heat transfer agents, power and consumables (e.g., filter membranes, chromatography resins) used in the analyses were determined using the SuperPro Designer built-in equipment cost model and default databanks. For the cellulase case study, the program's parameters such as water costs and total capital investment distributed cost factors were set to be the same as those used in the model described in Klein-Marcuschamer et al. [16]; this SuperPro Designer model is also available at the Joint Bioenergy Institute (JBEI) technoeconomic analysis wiki site (http://www.lbl.gov/tt/techs/lbnl2678.html).

Additional case study specific design parameters were selected based on experimental data from journal articles, patent literature, the authors' laboratory, interviews with scientists and technologists conducting the work cited, technical specification sheets or correlations, heuristics, or assumptions commonly used in the biotechnology and/or agricultural industry. The case study models were based on a new “greenfield” facility, operating in batch mode, although annual production costs neglecting the facility dependent costs were also determined to predict annual production costs using an existing facility. For the butyrylcholinesterase case study, annual operating time of 7920 hours (330 days, 24- hour operation, or 90% online) for the facility was used with indoor grown* Nicotiana benthamiana* plants. It was assumed that the plants would be grown continuously throughout the year (8760 hours, or 365 days, 24-hour operation, or 100% online). For the cellulase case study, since the tobacco plants are grown in the field, it is assumed that plant growth occurs for 215 days of the year (in North America, seeding begins at the end of March and final harvest is at the end of October; 59% online) and the indoor facility is in operation for 127 days per year (35% online). For comparative purposes in the cellulase case study, the laboratory/QA/QC costs were neglected since they were neglected in the JBEI model and such costs are likely to be a minor component for the industrial enzyme case study. The following items were also neglected in both case studies: land costs, upfront R&D, upfront royalties, and regulatory/certification costs as these can vary widely. SuperPro Designer files (^∗^.spf) for the case studies can be downloaded from http://mcdonald.ucdavis.edu/biologics.html, and require SuperPro Designer software to run/view. An evaluation (demo) version of the software can be downloaded from the website: http://www.intelligen.com/downloads.html to view and run the case study files. For the butyrylcholinesterase case study, the process flowsheet was split into separate modules to better understand the contributions of various process segments.

### 2.2. Modeling Protocol

Process flow and unit operations were derived from published methods and results from a number of sources as indicated in each case study, and from interviews with leading gene expression, agronomy, and manufacturing scientists and engineers who have participated in the development and scale-up of the processes described. On the basis of this information, the SuperPro Designer software was applied to calculate material inputs and outputs, bulk, and per-dose or per-unit costs.

### 2.3. Host Plant Species Selection and Justification

The two AI classes evaluated in these studies are produced in* Nicotiana* host plants.* Nicotiana* species, notably* N. tabacum, N. excelciana,* and* N. benthamiana,* are preferred hosts for PMB manufacture due to their metabolic versatility, permissiveness to the propagation of various viral replicons, and high expression yields achievable with a wide range of targets, as reviewed by Pogue et al. [19], De Muynck et al. [20], Thomas et al. [1], Gleba et al. [5], and others. Use of these hosts for production of clinical trial materials is also familiar to FDA and other regulatory agencies, thus facilitating* Nicotiana's* acceptance in regulation-compliant manufacturing [5, 21–24].

### 2.4. Modeling Production of Butyrylcholinesterase

#### 2.4.1. Product Selection and Justification

The enzyme is a globular, tetrameric serine esterase with a molecular mass of approximately 340 kDa and a plasma half-life (*t*_1/2_) of about 12 days; the plasma *t*_1/2_ is largely a function of correct sialylation [25, 26]. BuChE has several activities, including the ability to inactivate organophosphorus (OP) nerve agents before they can cause harm. With the recent use of chemical nerve agents such as sarin, there is continued interest on the part of many governments in stockpiling BuChE as a countermeasure. Currently BuChE is purified from outdated blood supplies; however, the high cost of this route (~$20,000 per treatment with 400 mg enzyme [27]) and its low supply limit its utility [28]. It has been estimated that extraction of BuChE from plasma to produce 1 kg of enzyme, which would yield small stockpile of 2,500 400-mg doses, might require extraction of the entire US blood supply [29]. Large amounts of the enzyme are required for effective prophylaxis because of the 1 : 1 enzyme/substrate stoichiometry needed for protection against OP agents. Not surprisingly, recombinant routes have been explored and the enzyme can in fact be produced by microbial fermentation [30], animal cell culture [31, 32], and transgenic goats [33] and stably or transiently expressed in* Nicotiana*, albeit at modest levels of 20–200 mg/kg fresh weight (FW) biomass [29, 34, 35], with yield improvements being the target of ongoing research. The bacterial product is nonfunctional and the mammalian cell culture products do not have the plasma *t*_1/2_ needed for prophylaxis and may be difficult and expensive to scale, as discussed by Huang et al. [33]. Goat-milk produced BuChE can be obtained at 1–5 g/L milk [33], but consists mostly of dimers, is undersialylated and has short plasma *t*_1/2_. While expression yields are impressive, transgenic animal sources face challenges of herd expansion to satisfy emergency demand, as well as potential adventitious agent issues, and these challenges need further definition. Furthermore, of these options, only plant-based biosynthesis yields an enzyme that is sialylated (as described below) and appears to reproduce the correct tetrameric structure of the native human form in sufficient yield to be commercially attractive [29, 36]; hence, the plant-based route became the basis for our modeling exercise. Not surprisingly, the plant route for BuChE manufacture is also the subject of continued DARPA interest and support [27, 37].

#### 2.4.2. Gene Expression Options

BuChE can be produced stably in recombinant plants or transiently in nonrecombinant plants by viral replicons delivered by agrobacterial vectors introduced into the plants via vacuum-assisted infiltration. Relative to stable transgenic plants, the advantages of speed of prototyping, manufacturing flexibility, and ease of indoor scale-up are clearly differentiating features of transient systems and explain why this approach has been widely adopted in the manufacture of many PMP (recently reviewed by Gleba et al. [5]). In our analysis of BuChE, we used expression yields from several sources that evaluated various* Agrobacterium*-mediated expression systems, including Icon Genetics' magnICON expression technology (“magnifection”) [29, 34–36]. Magnifection should be familiar to most readers of this volume as it has been applied in R&D programs throughout the world and its features have been the topic of multiple original studies and reviews (see, e.g., Marillonnet et al. [38]; Giritch et al. [6]; Gleba and Giritch [39], Klimyuk et al. [4], and Gleba et al. [5]); therefore, the method is not described here in further detail. Likewise, the process of vacuum-assisted infiltration has been described in detail by Klimyuk et al. [4], Gleba et al. [5], and others and is not further explained here.

#### 2.4.3. Plant Host and Upstream Process

For BuChE, we modeled the use of an* N. benthamiana* transgenic line modified to express the mammalian glycosylation pathway, beginning with a mutant host lacking the ability to posttranslationally add plant-specific pentoses (ΔXF) but with the ability to add galactosyl and sialic acid residues to polypeptides, based on work recently reported by Schneider et al. [36]. Use of this host obviates the need to enzymatically modify the plant-made polypeptide* in vitro* after recovery to ensure the presence of correct mammalian glycan, a procedure that could substantially increase the cost of the AI [40]. A glycan-engineered host can be produced in two ways, by stable transformation or via use of multigene agrobacterial vectors. The feasibility of sialylation via the latter approach was shown recently by Schneider et al. [36] for BuChE. Although there is an extra element of time required to develop a stable transgenic host compared to the transient modification of a pathway, the availability of a transgenic plant obviates the need to manufacture several* Agrobacterium* vectors carrying the genes for the product and two (or more) binary vectors carrying genes for the sialylation pathway; a procedure that would require additional capital and operational investments to generate multiple inocula in large scale. Therefore, for modeling upstream processes, we assumed that transgenic seed was available and that the resultant BuChE would have mammalian glycans and form tetrameric structures [29], and hence its biological activity and plasma half-life would be comparable to the native human enzyme [29, 36].

#### 2.4.4. Downstream Purification

**Figure 1:**
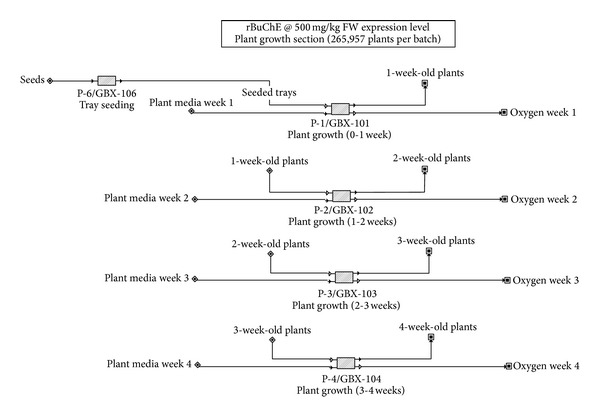
Indoor growth of* Nicotiana benthamiana* plants.

**Figure 2:**
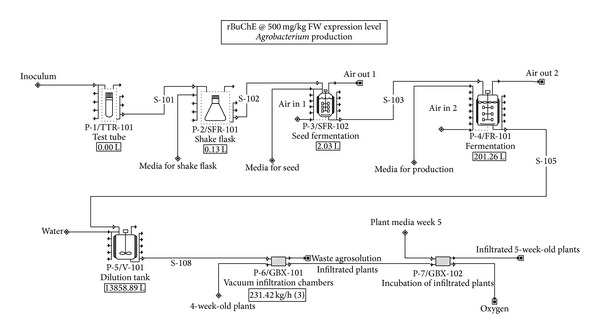
Agrobacterial growth, vacuum infiltration, and incubation.

**Figure 3:**
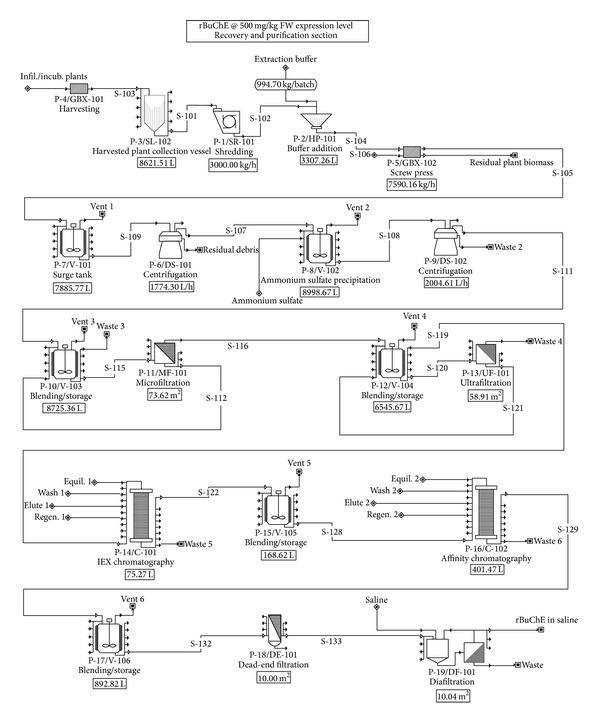
Downstream processing: recovery and purification of rBuChE.

To model downstream purification of BuChE, we assumed harvest and extraction at 7 days after inoculation. Biomass disruption was by homogenization, followed by filtration and clarification, as generally described [28, 34], but with modifications required for scale-up as indicated in Results and Discussion. Purification of the enzyme was by procainamide affinity chromatography [28]. In the overall process, plant growth, inoculation, and product accumulation steps occur indoors in controlled environments, and extraction, clarification, and final purification of BuChE take place in classified suites, so that manufacturing and release of the enzyme can be compliant with FDA cGMP guidance for human therapeutics. Design premises for this process, specific assumptions used in modeling, and resultant cost calculations are presented (see Section 3.1, Tables 1, 2, 3, and 4, and Figures 1, 2, and 3).

### 2.5. Modeling Production of Cellulases

#### 2.5.1. Product Selection and Justification

Cellulases currently under evaluation in bioethanol programs are all produced by microbial fermentation. Despite decades of research on lowering cellulase manufacturing costs, these enzymes still account for 20–40% of cellulosic ethanol production costs [41, 42]. Hence, lowering the cost of the biocatalyst is critical to the eventual adoption of biofuel processes that utilize renewable plant biomass feedstocks without competing with food or feed supplies. An alternative to fermentation-produced cellulases is the production of these enzymes in crop plants, with the ultimate goal of producing cellulases at commodity agricultural prices. This process concept was modeled to estimate enzyme and ethanol costs produced by this approach. Should such a process for cellulases prove economically viable, it might encourage the production of other cost-sensitive PMB as well as biomaterials, food additives, and industrial reagents.

#### 2.5.2. Gene Expression Options

Scale requirements and cost limitations of cellulases for biofuel applications constrained us to model production to open fields, with minimal indoor operations. We initially surveyed two scenarios for inducing production of cellulases in field-grown plants. The first was adaptation of the typical agroinfiltration method. Nomad Bioscience (Nomad Bioscience GmbH, Halle, Germany) has reported successful substitution of the agroinfiltration step with “agrospray,” a technique in which a suspension containing the* Agrobacterium* inoculant is admixed with a small amount of surfactant and sprayed onto the leaves of host plants [5, 43]. This approach eliminates the necessity to grow plants in containers (e.g., trays or carriers), a requirement imposed by the mechanics of the vacuum infiltration treatment in current procedures. Concomitantly, it also eliminates the cost of setting up and operating commercial-scale vacuum chambers, robotic tray manipulators, biomass conveyer systems, and so forth. Thus, this new approach should enable large-scale field inoculation of plants with agrobacteria and the production of biologics with more favorable economics. While we modeled the costs of producing cellulases via the agrospray approach, the sheer volume of enzymes needed for commercial-scale cellulosic ethanol processes necessitated a large investment in inoculum production infrastructure, including multiple fermentation trains and associated processing equipment. Further, the most efficient method of inoculating large areas was by aerial spraying, a procedure that not only entailed higher cost but that would also face regulatory uncertainties over spraying GM bacteria.

We opted instead for an alternative model using transgenic* N. tabacum* plants, each line of which carries an ethanol-inducible gene for one component enzyme of the cellulase complex. Synthesis of the cellulase is triggered by application of a dilute solution of ethanol (e.g., 2.5% v/v) onto the leaves, a process that has been demonstrated in small scale using a double-inducible viral vector [7]. We assumed that the dilute ethanol solution would be applied via ground irrigation systems that are currently used in agricultural practices, instead of aerial tankers. It was also assumed that the ethanol would be taken off as a side stream from the associated ethanol production facility that uses the cellulase enzymes. In so doing, we obviated the need to produce multiple inocula of GM bacteria and deliver them via aerial spraying. We were also able to model higher biomass density as well as higher expression yields of the enzymes* in planta*. These changes resulted in multiple economic benefits and were therefore adopted in our calculations.

#### 2.5.3. Plant Host and Upstream Process

Issues that are important in PMP, such as mammalian-like glycosylation or other posttranslational modifications, high purity, or specific formulation, are not relevant in the manufacture of cellulases and hence we modeled the use of conventional* Nicotiana* species in the production of the several enzymes necessary for complete saccharification of feedstock. The use of agricultural crops to produce enzymes at low cost has been suggested [5, 41]. In this case study, we modeled the use of stable transgenic* N. tabacum* varieties, each modified to express one cellulase protein upon induction with dilute ethanol. The process is based on inducible release of viral RNA replicons from stably integrated DNA proreplicons. A simple treatment with ethanol releases the replicon leading to RNA amplification and high-level protein production. To achieve tight control of replicon activation and spread in the noninduced state, the viral vector has been deconstructed, and its two components, the replicon and the cell-to-cell movement protein, have each been placed separately under the control of an inducible promoter [7]. In greenhouse studies, recombinant proteins have been expressed at up to 4.3 g/kg FW leaf biomass in the ethanol-inducible hosts [7], but seed lines for field application have yet to be developed. In our modeling, we assumed that each transgenic line would have been already field tested and available for implementation. We also assumed that large-scale stocks of each transgenic seed would need to be produced and have included this unit operation in our cost calculations.

Because cellulases are needed in different ratios to effect saccharification of different feedstocks, we assumed that seeds would be mixed at the appropriate ratios (considering expression levels in each host) and that the seed mixtures would be planted directly in the field. At maturity, what one would expect is a field of plants representing all the needed cellulase classes in the appropriate ratio for the intended feedstock. The current method of hydroponic cultivation of seedlings for transplantation to open fields, a common commercial tobacco cultivation practice to ensure germination and plants with good leaf size and quality, was substituted by direct seeding for more favorable economics. For example, traditionally tobacco may be grown at 12,000–16,000 plants/ha depending on variety [44, 45]. Higher-density seedling production for nontraditional uses of tobacco has been reported, targeting planting densities of over 86,000 plants/ha [44]. While transplanting ensures germination and quality, there is an economic limit to the scale at which it can be deployed with highly cost-sensitive AI, leading to interest in direct seeding practices. Experimental high-density cultivation studies via direct seeding have reported 400,000 to over 2 million plants/ha and biomass yields exceeding 150 mt/ha [46–48]. Our modeling included these higher-density practices to determine economic impact.

#### 2.5.4. Downstream Recovery

**Figure 4:**
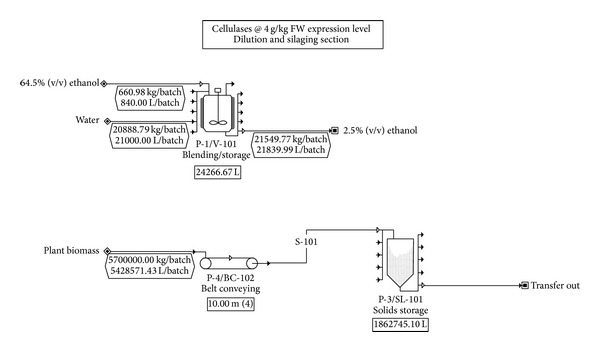
Process operations in the manufacture of cellulases in tobacco biomass.

**Figure 5:**
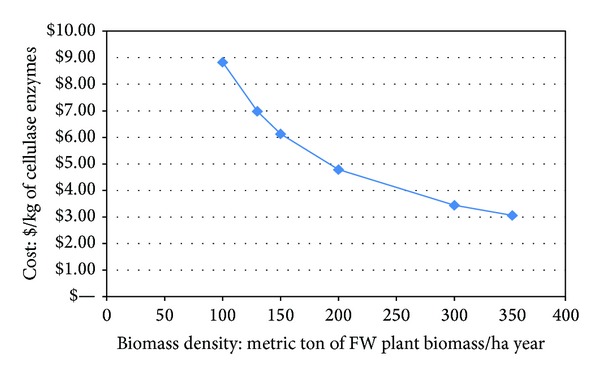
Cost of cellulase enzymes as a function of plant biomass.

In contrast to typical PMP products, the cellulases would not be extracted after accumulation; rather, the plants would be mechanically harvested and transported to a centralized facility for silaging and storage. Since the cellulase enzymes need to be continuously supplied to the saccharification process in the bioethanol plant and the harvested tobacco is only available for a limited period during the year, the silage inventory would increase during the tobacco-harvesting period and would decrease during the fall/winter. Cellulase activity in the ensilaged biomass is expected to be stable during the off-season storage [43]. For feedstock conversion, cellulase-containing biomass would be mixed with pretreated lignocellulosic feedstock (corn stover in our model) under controlled conditions to effect saccharification. Although not considered in this economic analysis, this feedstock replacement could also reduce corn stover feedstock requirements and associated costs. After separation of solids, the sugar solution would be fermented conventionally into ethanol, followed by distillation. The overall process we modeled is based on the US National Renewable Energy Laboratory (NREL) process described by Humbird et al [49], with substitution of fungal cellulase production in the NREL model by the cellulases stored as silage described herein. Design premises for this process, specific assumptions used in modeling, and the resultant cost calculations are presented (see Section 3.2, Tables 5 and 6, and Figures 4 and 5).

## 3. Results and Discussion

### 3.1. Butyrylcholinesterase Process Design Premises and Assumptions

The following premises and assumptions were used for evaluation of rBuChE biomanufacturing (Table 1). Although we calculated the construction of a new dedicated manufacturing facility, we also calculated operating costs if a facility with the required capacity were to be already available for toll-manufacturing of the enzyme; results are reported for both scenarios. The overall process is broken into three components: (1) indoor growth of* Nicotiana benthamiana* (Figure 1); (2)* Agrobacterium* growth, vacuum infiltration, and* N. benthamiana* incubation (Figure 2); and (3) rBuChE recovery and purification (Figure 3).  Table 2 shows the capital cost adjustment factors used for each section of the facility.

Process flowsheets for rBuChE production are shown. The seeding and indoor growth of* N. benthamiana* is shown in Figure 1. Each batch of plants (~266,000 plants comprising 1,039 4 ft × 4 ft trays per batch) will be grown indoors under LED lighting for 4 weeks prior to vacuum infiltration. Figure 2 shows the agrobacterial seed train and production fermentor (200 L with 160 L working volume), the vacuum infiltration system (3 vacuum chambers, each 6 ft diameter × 30 ft length), and the plant incubation facility for the infiltrated plants (6.8 days). The oxygen output streams indicated in Figures 1 and 2 represent net oxygen production by the plants due to photosynthesis. However, oxygen production was not included as part of the model since it does not impact the economics of the process. Figure 3 shows the downstream processes for recovery and purification of the rBuChE, which was modeled after the rBuChE lab purification scheme from vacuum infiltrated* N. benthamiana* described by Hayward [34] and the purification methods described by Lockridge et al. [28]. Major operations include plant harvesting, shredding, screw press/disintegration, ammonium sulfate precipitation, centrifugation, tangential flow microfiltration, tangential flow ultrafiltration, ion exchange chromatography, affinity chromatography, and diafiltration.

#### 3.1.1. Manufacturing and Economic Calculations

Table 3 shows the total capital investment and annual operating costs for the plant-made rBuChE facility at an expression level of 500 mg/kg FW plant biomass (vacuum infiltration of 4-week old plants and 7 days after infiltration). The annual operating costs are shown with and without facility dependent costs (e.g., depreciation) to simulate a new facility and use of an existing facility, respectively.  Table 4 shows the resulting rBuChE cost per dose for both cases.

Table 3 shows the breakdown of the capital investment and operating costs for the plant-made rBuChE and indicates that the unit production costs are estimated to be about $234/dose if facility dependent costs are not included in the annual operating costs or about $474/dose if these costs are included. Most of the capital cost (~60%) and a significant portion of the operating costs (>70–75%) are associated with the recovery and purification of rBuChE. Our base case assumed rBuChE expression of 500 mg/kg FW because that is a target expression level in ongoing research at several institutions. If a currently achievable level of 100 mg/kg FW is used instead (reported expression range is 20–200 mg/kg FW [29, 34, 35]), the costs increase to $1,210/dose and $430/dose when including and excluding facility dependent costs, respectively. In any scenario examined, the production costs in plants are significantly lower than the estimated production costs for blood-derived BuChE (~$10,000/dose).

We recognize that additional modification or formulation of the plant-produced enzyme might be necessary or desirable prior to adoption for human use and that such additional modifications would increase the cost of the AI. For example, Geyer et al. [29] reported improved pharmacokinetics of PEGylated plant-produced BuChE relative to the nonmodified enzyme. However, because consensus on the preferred options for modification has not yet been reached, we omitted these additional steps from our calculations.

### 3.2. Cellulases Process Design Premises and Assumptions

The following premises and assumptions were used for evaluation of cellulase biomanufacturing in open fields. Due to the fact that this process is specialized and due to the scale and input requirements of a modern biofuels operation, our analysis included the construction of a new, dedicated manufacturing facility to provide the required cellulase enzymes for a large-scale (61 million gallons per year) cellulosic ethanol facility (Table 5).

Figure 4 shows the process operations required for cellulase enzyme production on a per-batch basis. The flowsheet on the top shows the blending tank needed for preparation of the ethanol induction solution to be applied in the field, and the flowsheet on the bottom shows the transport and storage operations following harvest of the transgenic tobacco.

#### 3.2.1. Manufacturing and Economic Calculations

Table 6 shows the total capital investment and annual operating costs for the production of 2.87 million kg of cellulase enzymes per year (unpurified) at an expression level of 4 g cellulase/kg FW tobacco biomass and a plant density of 130 metric tons of biomass per hectare per year. The table also indicates the corresponding costs obtained from the JBEI model for fungal fermentation-based production of approximately the same amount of cellulase enzymes per year (2.82 million kg cellulases/year).

For the base case study, the plant-based system results in a >30% reduction in unit production costs for the cellulases as well as an 85% reduction in the required capital investment. For the plant-based cellulase production system, the major contributors to the unit production cost were the costs associated with tobacco cultivation (70%), the costs associated with ethanol spraying (20%), followed by the costs associated with ethanol dilution, transporting and storage (8%), and seed costs (4%). The differences in total capital investment and annual operating costs for the two cellulase production platforms are not surprising, since the fungal fermentation area alone requires twelve 288,000-L fermenters along with the seed train necessary to provide the inoculum for the production fermenters. The differences between the two systems would be expected to be even larger if the total capital investment included additional factors for associated piping, instrumentation, insulation, electrical facilities, buildings, yard improvements, and auxiliary facilities (these were not included in the plant-based model since they were neglected in the JBEI model) because these would be reflected in the facility dependent component of the annual production costs.

Figure 5 shows the effect of biomass density on the unit production costs for cellulase enzyme using the ethanol-induced tobacco system and indicates, as expected, that the cost of goods decreases as tobacco biomass density increases. In agronomic studies with field-seeded tobacco cultivated at high density, biomass yields exceeding 150 mt/ha have been achieved [47, 48]; higher field densities may be possible with selected varieties and specialized agronomic practices.

## 4. Conclusions

With hundreds of candidate biologics in development, traditional protein-manufacturing practices may face a major global capacity shortage for the production of new and off-patent biotherapeutics. Worldwide, there are approximately three dozen facilities capable of very large-scale biotherapeutics manufacturing; thus, traditional methods may not produce sufficient quantities of products to meet patient population needs. The challenge is compounded when food additives, industrial products, and biomaterials are added to the capacity estimates. The addition of plants as a biomanufacturing platform could help alleviate this shortage.

Several advantageous features of plant-based systems continue to support interest in plant-based PMB manufacturing. Among them is the potential to scale upstream expression with considerably lower capital requirements compared to traditional cell culture processes. Plant viral replicons delivered within agrobacterial vectors have shown superior speed relative to transgenic plants and proven robust when scaled to industrially relevant settings [2, 4–6, 20, 23]. Conversely, if a large and continuous supply of a consistent AI is needed at low cost for industrial applications, the use of inducible promoters in transgenic plants grown in the field can offer advantages in obviating the production of very large volumes of agrobacterial inoculum and its application over large areas [5, 7]. Plants are also free of adventitious agents that can infect humans and animals (a concern in cell-based systems and transgenic animals) and this inherent safety feature pays dividends by enabling the streamlined purification of the final product without the need for adventitious agent removal steps. Plants' eukaryotic protein processing enable them to synthesize complex classes of biomolecules, such as monoclonal antibodies, therapeutic enzymes, and multiepitope vaccines that are at the forefront of pharmaceutical interventions. Recent advances in glycoengineering of host plants have enabled the production of human- and mammalian-identical (or at least mammalian-similar) molecules that exhibit comparable or even superior pharmacology to their cell culture-derived counterparts [5, 50, 51]. Inescapably, the growth of the population in developing world regions, the aging of the population in industrialized countries, population displacement due to political turmoil, degradation of environmental quality, and depletion of nonrenewable resources are serious challenges that have not been and likely cannot be readily met only by the existing product manufacturing platforms. This creates new opportunities for plant-based systems to yield lower cost and more widely accessible biopharmaceuticals, food, feed, fuels, and industrial materials.

Here we analyzed the technoeconomics of plant-based manufacture for two active ingredients, both of them enzymes, under development for widely different markets: butyrylcholinesterase for use as a medical countermeasure and a cellulase complex for the production of cellulosic ethanol. In the first case study on BuChE, we modeled transient vectors encoding the protein of interest introduced into glycan-engineered* N. benthamiana* host plants via vacuum-assisted agroinfiltration, followed by disintegration of the plant biomass and extraction and purification of the AI. This route was taken because we anticipate exploratory modifications to the composition of the AI during its development cycle, and transient expression enables the most facile and economic prototyping of the product with direct scalability to commercial production. In contrast, in the second case study on cellulases, we modeled the use of transgenic host plants carrying the genes for each of the enzymes in the cellulase complex under the control of an ethanol-inducible promoter element. Harvest of the biomass is followed by partial drying to produce silage without further purification of the AI. This route was taken to obviate the cost of inoculum manufacture and aerial application, considering the vast areas of land that would need to be dedicated to cellulase biosynthesis. The penalty we accepted is the time to develop each transgenic line.

In both evaluations, we applied the SuperPro Designer modeling tool to generate discrete input and output data for each unit operation, from which we derived bulk AI as well as per-unit/per-dose costs. The calculated costs for these products made in plants were compared to publicly available costs for the same AI produced through predecessor technologies.

### 4.1. Butyrylcholinesterase

With the assumptions and process parameters adopted for this case study, our results show that rBuChE could be manufactured in plants using transient expression for approximately $234 per 400-mg dose if an existing toll-manufacturing facility were available to accommodate production of 25 kg/year of purified enzyme (equivalent to 62,500 doses/yr). If a new facility with that capacity needs to be built, the cost per dose is projected to increase to approximately $474. Further economic gains could be possible if capacity were to be increased to 100 kg of enzyme per year or more (data not shown), which, in a toll-manufacturing scenario, could reduce the cost of rBuChE to below $200/dose. Even with conservative assumptions, these costs are dramatically below the costs obtainable with blood-extraction processes for this enzyme and may be substantially lower than those for transgenic approaches. In addition, the combination of speed of product prototyping enabled by transient expression, the superior quality and functionality of the rBuChE obtained, lack of adventitious agents, and the rapid scalability of plant systems should make plants the preferred platform for the rapid and cost effective production of this and similar products.

### 4.2. Cellulases

With the assumptions and process parameters adopted for this case study, our results show that high-density field cultivation of tobacco induced to synthesize several enzymes of the cellulase complex could be competitive with fungal cellulases produced by fermentation for the saccharification of biomass in the production of cellulosic ethanol. Our model adopted many of the process parameters from published studies on the conversion of lignocellulosic feedstocks (in our case corn stover); we replaced the unit operations for the fungal-sourced enzymes with the unit operations for the plant-sourced catalyst and compared operating costs and cost per kg of cellulase blend. Using 130 mt/ha of transgenic tobacco biomass as our base case, our model suggests that plant-sourced cellulases could be produced for just under $7/kg. Even when using a more conservative biomass yield of 100 mt/ha, plant-sourced cellulases could be produced for under $9/kg. These costs compare favorably to the more than $10.6/kg for the fungal-sourced product (all costs adjusted to 2013 US dollars). In a high-volume industry such as biofuels manufacturing, these differences would be significant. These estimates could change depending on how closely empirical results from field trials compare to the modeled assumptions (e.g., expression yields in* N. tabacum* or* N. excelciana* versus those in* N. benthamiana*; length of growing season; weather and other environmental variables, etc.). Conversely, because some of the process assumptions were derived from nonoptimized pilot studies, significant further improvements might be possible in agronomic output, gene expression yield, and cellulase processing efficiency, potentially resulting in even more favorable economics for cellulases and other cost-sensitive, high-volume PMIP.

### 4.3. Concluding Remarks

The SuperPro Designer modeling software used in these case studies accommodated all major process unit operations in two widely different PMB manufacturing approaches. The program is flexible and allows adaptation through user-definable functions to complement its existing equipment and cost database. Future work will include refinement of the model with specific focus on PMP/PMB/PMIP unit operations and application of the refined model to technoeconomic studies of other plant-made products. It is our hope that wider adoption of evaluations such as the ones presented here will assist decision-makers in early stage product target selection. Doing so would enable the best match to be found between a product's features and its preferred manufacturing platform early enough in the process to avoid costly mistakes in later stages of development.

## Figures and Tables

**Table 1:** Recombinant butyrylcholinesterase (rBuChE) design premises and key assumptions.

Parameter	Value
General assumptions for the facility	
rBuChE production level	25 kg rBuChE/year (bulk)
rBuChE doses per year	62,500 doses/year at 400 mg/dose
Downstream Recovery/purification yield	20%
Annual operating days	330 days
rBuChE production in plants following infiltration/incubation	125 kg rBuChE/year
Batch cycle time (time between start of new infiltration batches)	7 days
Batches per year	47
Base case rBuChE expression level	500 mg/kg FW at ~7 days after infiltration
Facility lifetime	15 years
Depreciation	Straight line over 10 years, 5% direct fixed capital salvage
Working capital	30 days of labor, materials, utilities, waste treatment
Lab/QC/QA costs	2% of total labor costs for plant growth and agroinfiltration sections, 15% of total labor costs for recovery and purification section
Start-up/validation costs	5% direct fixed capital

Assumptions for indoor plant growth section	
Mass per plant at 5 weeks	0.02 kg FW/plant
Tray size	4 ft × 4 ft
Number of plants per tray	256
Tobacco seed cost	$0.001/seed
Age at infiltration	4 weeks
Total number of plant batches in inventory	5 batches (just seeded, 1 wk old, 2 wk old, 3 wk old, 4 wk old—ready to infiltrate, 5 wk old—infiltrated, incubated and ready to harvest)
Total number of plants in inventory	~1.3 million
Total plant growth area	83,320 ft^2^ total, or 10 levels with 8,332 ft^2^ footprint
LED fixture costs	$40/ft^2^ plant growth area (including capital cost factors for plant growth area as shown in Table 2)
LED energy costs	20 W/ft^2^

Assumptions for *Agrobacterium* production, vacuum infiltration, and plant incubation section	
Agro “loading”—mass of recombinant *Agrobacterium* to mass of plant tissue	0.00001 kg dry weight (dw) bacteria/kg FW plant biomass
*Agrobacterium* biomass density at 12 hours of culture	2.6 g dw/L = 0.0026 kg dw/L
Inoculum density used in seed train	1% V/V
Dilution factor between agroinfiltration solution and agrobacterial production fermentor	78
Percent weight change in plant tissue following vacuum agroinfiltration	30%
Trays processed per vacuum chamber (30 ft) per day	336 trays/chamber/day
“Excess” *Agrobacterium* solution used	87% of total infiltration solution
Incubation time for infiltrated plants	~7 days

Assumptions for the rBuChE recovery and purification section	
Overall yield in downstream processes	20%
Harvesting rate	3 trays/minute
IEX chromatography	
Binding capacity	20 mg/mL
Resin cost	$1,839/L
Number of reuse cycles	100
Affinity chromatography	
Binding capacity	3 mg/mL
Resin cost	$10,000/L
Number of reuse cycles	30

**Table 2:** Capital cost factors for rBuChE case study.

Capital cost factors						
Estimated based on PC (listed equipment PC plus unlisted equipment PC)						
Unlisted equipment	0.2 listed purchased equipment cost					

Direct costs	Plant growth		Agroproduction/infiltration		rBuChE recovery/purification	

Piping	0.1	PC	0.35	PC	0.35	PC
Instrumentation	0.2	PC	0.4	PC	0.4	PC
Insulation	0.01	PC	0.03	PC	0.03	PC
Electrical facilities	0.1	PC	0.1	PC	0.1	PC
Building	0.2	PC	0.45	PC	3	PC
Yard improvement	0.15	PC	0.15	PC	0.15	PC
Auxiliary facilities	0.1	PC	0.4	PC	0.4	PC
UE installation	0.5	UEPC	0.5	UEPC	0.5	UEPC
**Direct costs multiplicative factor**	**2.35**		**2.88**		**5.43**	
Engineering	0.25	DC	0.25	DC	0.25	DC
Construction	0.35	DC	0.35	DC	0.35	DC
**Indirect costs multiplicative factor**	**1.41**		**1.73**		**3.26**	
Contractors fee	0.05	DC + IC	0.05	DC + IC	0.05	DC + IC
Contingency	0.1	DC + IC	0.1	DC + IC	0.1	DC + IC
**Other costs multiplicative factors for DFC**	**0.56**		**0.69**		**1.30**	
**Total multiplicative factor for DFC**	**4.33**		**5.30**		**9.99**	
**Total multiplicative factor for TCI (except working capital)**	**4.54**		**5.56**		**10.49**	

PC: purchase cost; DC: direct cost; IC: indirect cost; UEPC: unlisted equipment purchase cost; DFC: direct fixed capital; TCI: total capital investment.

**Table 3:** rBuChE facility cost summary (in millions of US dollars).

	Plant growth	*Agrobacterium* inoculum growth/infiltration/incubation	Recovery/purification	Totals
Total capital investment	$16.1	$19.6	$56.7	$92.4
Annual operating costs **excluding** facility dependent costs	$2.8	$0.89	$10.9	$14.6
Annual operating costs **including** facility dependent costs	$4.3	$4.5	$20.7	$29.5

**Table 4:** rBuChE production cost summary (in US dollars).

	Plant growth	*Agrobacterium* inoculum growth/infiltration/incubation	Recovery/purification	Totals
Cost per dose **excluding** facility dependent costs	$45	$14	$175	$234
Percentage of cost	19.2	6.0	74.8	100.0
Cost per dose **including** facility dependent costs	$70	$72	$332	$474
Percentage of cost	14.8	15.2	70.0	100.0

**Table 5:** Open field cellulase manufacturing design premises and key assumptions.

Parameter	Value	Source
Cellulosic ethanol facility assumptions		
Cellulosic ethanol facility capacity	61 million US gallons/year	Humbird et al., 2011 [49]
Cellulosic feedstock	700,000 metric tonnes (dry) corn stover per year, 2,000 metric tonnes/day	Humbird et al., 2011 [49]
Land area required for corn stover feedstock	2,034,000 hectares/year	Humbird et al., 2011 [49]
Annual operating hours	8,410 hours/year	Humbird et al., 2011 [49]
Conversion	87 gallons ethanol/metric tonne corn stover @76% conversion	Humbird et al., 2011 [49]
Enzyme loading	20 mg enzyme “protein mixture” per gram cellulose in feedstock (2% wt/wt)	Humbird et al., 2011 [49]
Enzyme mixture required	4,100,000 enzyme “protein mixture”	Humbird et al., 2011 [49]
Cellulase enzyme required	2,870,000 kg/year	About 2% higher than in Klein-Marcuschamer et al., 2012 [16]
Cellulase in enzyme mixture	70.0% total soluble protein	Calculated from above

Base case tobacco agronomic and cellulase enzyme production assumptions		
Base case tobacco biomass production	130 metric tonne fresh weight (FW)/ha year	
Mass of a full grown tobacco plant growth at high density	1.0 kg fresh weight (FW)/plant	
Number of plants per hectare	130,000 plants/hectare	Calculated
Tobacco seed cost	$0.001/seed	
Tobacco growth cycle	82 days from seed to induction, harvest at 7 days after induction spray	
Tobacco planting season (US Midwest/South)	Late March to late October	
Land reuse during growing season	Based on a total of 127 plant batches per year, land recycling can start with batch 94, so land requirement is only 0.74 of that required if no land was reused	Calculated
Tobacco production cost (labor and machinery for seeding, harvesting)	$1,000/hectare	
Number of tobacco batches per year	127 batches/year	Calculated
Cellulase expression level	4 g cellulase/kg FW tobacco at 7 days after induction, with 2 applications of ethanol induction solution	Werner et al., 2011 [7]
Land area required	5,519 hectares/year	Calculated
Tobacco land area required as a fraction of corn stover land area required	0.27%	Calculated
Ethanol induction	Foliar application of aqueous solution of 2.5% (v/v) ethanol, 2 applications using ground irrigation/sprinklers (central pivot, traveler, or side roll), at 500 L/hectare	
Cost of ethanol for induction	$0.73/kg	Humbird et al., 2011 [49]
Percentage of ethanol drawn off from biorefinery for induction	0.12%	Calculated
Capital cost of ethanol spray irrigation system	$2,223/hectare	http://www.nrcs.usda.gov/Internet/FSE_DOCUMENTS/nrcs141p2_023892.pdf
Annual operating cost of ethanol spray irrigation system	$988/hectare year	http://www.caswcd.org/Irrigation%20guide/Sec7.pdf

Base case assumptions for plant-based cellulase production facility		
Construction period	12 months	Klein-Marcuschamer et al., 2012 [16]
Start-up period	18 months	Klein-Marcuschamer et al., 2012 [16]
Project lifetime	25 years	Klein-Marcuschamer et al., 2012 [16]
Income tax rate	40%	Klein-Marcuschamer et al., 2012 [16]
Working capital	30 days of labor, raw materials, utilities, waste	Klein-Marcuschamer et al., 2012 [16]
Start-up cost	5% direct fixed capital investment, not depreciable	Klein-Marcuschamer et al., 2012 [16]
Depreciation	Straight line over 10 years, salvage value 5% direct fixed capital	Klein-Marcuschamer et al., 2012 [16]
Unlisted equipment	5% of the major purchased listed equipment	Klein-Marcuschamer et al., 2012 [16]

**Table 6:** Capital investment and operating costs for manufacturing of cellulases in field-cultivated plants (in 2013 US dollars).

	Plant-based cellulase production process	Fungal-based cellulase production process
Total capital investment (millions of US dollars)	$11.5	$81.5
Total annual operating costs per unit of cellulase production (millions of US dollars)	$20.0	$29.9
Unit production cost ($/kg cellulase)	$6.98	$10.6
